# Expression of Maspin and Ezrin Proteins in Periocular Basal Cell Carcinoma

**DOI:** 10.1155/2014/596564

**Published:** 2014-12-15

**Authors:** Mansooreh Bagheri, Masoomeh Eghtedari, Mandana Bagheri, Bita Geramizadeh, Mohammadreza Talebnejad

**Affiliations:** ^1^Poostchi Ophthalmology Research Center, Shiraz University of Medical Sciences, Shiraz, Iran; ^2^Ophthalmology Department, Shiraz University of Medical Sciences, Shiraz, Iran; ^3^Pathology Department, Liver Transplant Research Center, Shiraz University of Medical Sciences, Shiraz, Iran

## Abstract

*Background.* The aim of this study was to investigate maspin and ezrin expression in different subtypes of periocular basal cell carcinoma (BCC). *Methods.* Tissue samples from 43 patients with periocular BCC. Our cases were comprised of 10 morpheaform, 25 nodular, and 8 adenoid type BCCs. Immunohistochemical staining for maspin and ezrin was performed by Envision detection system. *Results.* There was no difference between different subtypes of BCC in maspin expression regarding positivity, intensity, and pattern of expression. Ezrin was expressed in all subtypes of BCC but the intensity was significantly higher in morpheaform BCC compared to nodular and adenoid types (*P* < 0.001 and *P* = 0.012, resp.); ninety percent of morpheaform samples showed strong ezrin intensity, while this strong intensity was only present in 25% and 12% of adenoid and nodular subtypes, respectively. There was no correlation between age, sex, or tumor margin involvement and expression of neither maspin nor ezrin. There was no correlation between maspin and ezrin expression except in nodular type, in which an inverse correlation was found (*P* = 0.004). *Conclusion.* Ezrin is expressed intensely in morpheaform BCC of periocular region. Further studies are needed to show the significance of this finding in prognosis of morpheaform BCC.

## 1. Introduction

Basal cell carcinoma (BCC) of the skin, the most frequent malignancy in human population, represents 20% of eyelid tumors and 90% of eyelid malignancies [1, 2]. BCC subtypes, including nodular, adenoid, superficial, micronodular, and morphoeic/infiltrative subtypes, have different clinical and morphological pictures [3].

Recently, various tumor biomarkers are identified which have great importance in predicting clinical behavior of the cancers [3], among them, maspin and ezrin may be involved in BCC pathogenesis. Maspin protein, a member of the serpin family of protease inhibitors, presents as a secreted, cytoplasmic, nuclear, or cell surface-associated protein [4, 5]. Maspin is the product of a tumor suppressor gene and is involved in apoptosis and inhibition of carcinoma invasion, metastasis, and angiogenesis [4]. Its expression is downregulated during cancer progression [6].

Ezrin, a member of the ERM (ezrin-radixin-moesin) protein family, acts as linkers between the cell membrane and the actin cytoskeleton and is involved in several cellular functions, including cell adhesion to the extracellular matrix, cell-cell communication, signal transduction, and apoptosis [7, 8]. Ezrin has active role in regulating tumor growth and progression and metastatic dissemination of many cancers [9, 10].

Little is known about expression of maspin and ezrin biomarkers in periocular skin tumors. The aim of this work was to investigate maspin and ezrin expression in periocular BCC to throw light on their role in pathogenesis of this carcinoma by immunohistochemistry, together with correlating their expression with the clinicopathological features of the tumor.

## 2. Materials and Methods

Excised tissue samples, obtained from 43 patients with diagnosis of periorbital BCC, were retrieved from archive of Pathology Laboratory at Khalili Hospital, Shiraz University of Medical Sciences, during April 2011 to April 2012. All patients were diagnosed initially during this period and no patient received any treatment for their BCC prior to sample collection. Hematoxylin & eosin stained sections were examined under the light microscope for confirmation of the diagnosis and determination of BCC type and involvement of tumor margins. Metatypical carcinomas with squamous differentiation in histological evaluations were excluded. Tissue samples from pigmented BCC cases showed pathologic characteristics of nodular type, so they were assigned as nodular type. Five micrometer-thick sections were taken from paraffin-embedded tissue blocks and mounted on poly L lysine slides. Then sections were deparaffinized in xylene and rehydrated in descending grades of ethanol.

Immunohistochemical staining for maspin and ezrin was performed by Envision detection system. This is a 2-step procedure; the first step is incubation of the tissue with optimally diluted primary antibody (1/2000), and the second step is incubation of tissue with Envision reagents. Envision reagent is a peroxidase-conjugated polymer, which also carries antibodies to the rabbit or mouse immunoglobulins.

Maspin monoclonal primary antibody (mouse antihuman antibody, Santa Cruz Biotechnology Inc., Texas, USA) was raised against recombinant protein corresponding to N-terminal region of human maspin. Ezrin polyclonal primary antibody (rabbit antihuman antibody, Texas, Santa Cruz Biotechnology Inc., Texas, USA) was raised against C-terminus peptide of human ezrin. Maspin and ezrin antibodies were diluted to 1 : 2000 by TRIS-EDTA and citrate buffer, respectively. Antigen retrieval was done by boiling mounted tissue in TRIS-HCL buffer (pH 7.4). Then, primary antibody was employed and samples were kept overnight in 4°C.

Envision detection system consists of a dextran backbone coupled with peroxidase molecules and secondary antibody. The applied secondary antibodies (Cat number K5007, code number S3245 DAKO Cytomation) were mouse and rabbit IgG antibodies against maspin and ezrin, respectively. The substrate system was diaminobenzidine (DAB) chromogen. Mayer's hematoxylin was used as the counterstain. Prostatic glandular basal cells and duodenal mucosal tissue were used as the positive controls for maspin and ezrin, respectively. Normal skin was also used as positive control during staining for both maspin and ezrin. Negative control for staining of both maspin and ezrin was obtained by substitution of primary antibody with PBS in staining procedure.

Maspin immune reactivity was evaluated in tumoral tissue and adjacent epidermal layer as negative or positive, where the positivity was assigned as cytoplasmic and/or nuclear staining. Ezrin immunoreactivity was assessed in the tumor and adjacent epidermis and peritumoral lymphocytes (a reference of strong immunoreactivity). Cases were assigned positive for ezrin expression when cytoplasmic positivity with or without membranous immunoreactivity was present. The intensity of maspin and ezrin expression was assigned as a score of 0–3 with 0 indicating no staining and 1 for weak, 2 for moderate, and 3 for strong staining. All slides were independently assessed by two pathologists.

### 2.1. Statistical Analysis

Results were analyzed by statistical package SPSS version 17 (SPSS Inc. Chicago, USA). Fisher's exact test and Spearman correlation coefficient were used to analyze data. *P* value of <0.05 was considered statistically significant. Kappa statistic was used to test interrater reliability.

## 3. Results

**Figure 1:**
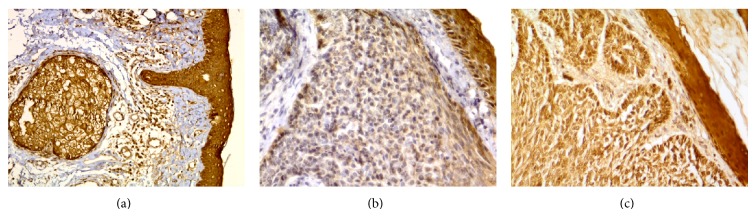
Maspin immunoreactivity in periocular basal cell carcinoma (immunohistochemical staining ×400 for (a), (b), and (c)): (a) strong maspin immunoreactivity in the epidermis and sebaceous gland in one of the control cases, (b) weak maspin immunoreactivity, and (c) moderate maspin immunoreactivity.

Our cases were comprised of 10 morpheaform, 25 nodular, and 8 adenoid type BCCs. No superficial, nodulocystic, and micronodular types were detected. All BCC samples along with control samples from normal skin showed diffuse cytoplasmic maspin expression in all epidermal layers. Clinical and pathological data of different types of periocular BCC are shown in Table 1. Maspin protein was expressed in 74.4% of samples. There was no significant difference between different types of BCC regarding maspin expression and intensity of staining (*P* = 0.63 and 0.82, resp.). Pattern of maspin expression was not different in BCC subtypes.  Table 2 shows maspin expression in different types of periocular BCC. Samples of negative, weak, and moderate maspin staining in tumoral cells are shown in Figure 1.

**Figure 2:**
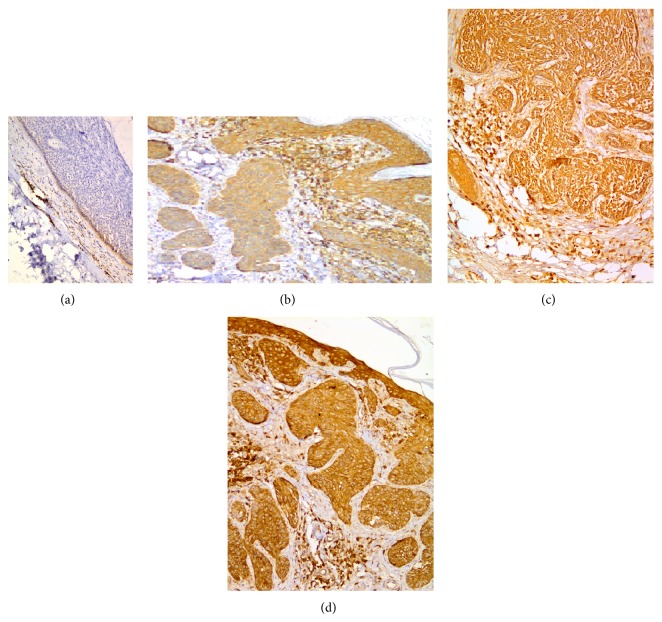
Ezrin immunoreactivity in periocular basal cell carcinoma (immunohistochemical staining ×200 for (a), ×100 for (b), ×200 for (c), and ×100 for (d)): (a) negative ezrin immunoreactivity, (b) weak ezrin immunoreactivity, (c) moderate ezrin immunoreactivity, and (d) strong ezrin immunoreactivity. Note strong immunoreactivity of tumor-associated lymphocytes and/or epidermis.

Ezrin protein was expressed in 93% of all samples. There was no significant difference between different types of BCC regarding ezrin expression while the intensity of staining was different among different types. Intensity of ezrin expression was significantly higher in morpheaform BCC compared to nodular and adenoid types (*P* < 0.001 and *P* = 0.012, resp.); ninety percent of morpheaform samples showed strong ezrin intensity, while this strong intensity was only present in 25% and 12% of adenoid and nodular subtypes, respectively. There was no significant difference between nodular and adenoid subtypes regarding ezrin intensity (*P* = 0.66). Ezrin expression among different types of periocular BCC is shown in Table 3.  Figure 2 shows samples of weak, moderate, and strong ezrin expression in tumoral cells.

There was no correlation between ezrin and maspin expression regarding intensity in morpheaform and adenoid BCC types (*P* = 0.20 and 0.16, resp.) but there was an inverse correlation between ezrin and maspin expression in nodular type (*P* = 0.004).

There was no correlation between age and expression of neither maspin (*P* = 0.78) nor ezrin (*P* = 0.75). We also did not find any significant correlation between margin involvement and maspin or ezrin expression (*P* = 0.12 and 0.058, resp.).

Sex did not have significant association with neither maspin nor ezrin expression (*P* value = 0.53 and 0.96, resp.). There was no significant association between sex and maspin and ezrin expression in any of the BCC subtypes.

We reached the Kappa value of 0.86 and 0.75 in maspin and ezrin assay for interrater reliability, respectively. Both values are in the range of almost perfect agreement. This finding means that the assay for maspin and ezrin was reproducible in this study.

## 4. Discussion

Identification of biomarkers, involved in the mechanisms of malignant cell transformation, is of great importance in predicting further clinical behavior of the cancer and has prognostic value in diagnosis and treatment [3]. In this study, we evaluated the expression of maspin and ezrin in different types of periocular BCC. Micronodular, infiltrative, basosquamous, morpheaform, and mixed BCC subtypes are known to have aggressive histological characteristics and adenoid variant is believed to be the nonaggressive subtypes [11]. We excluded metatypical BCC cases due to their intermediate features with squamous cell carcinoma. We did not detect micronodular, nodulocystic, and superficial types in our cases, so we categorized the cases to three groups: morpheaform, nodular, and adenoid.

Maspin, a product of tumor suppressor gene, is thought to inhibit carcinoma invasion, metastasis, and angiogenesis [12]. However, both decrease and increase of the expression of maspin parallel tumor progression [12]. Positive maspin expression was identified in 74.4% of our cases. Our results are in agreement with the study done by Reis-Filho et al. [13] in which maspin expression in BCC was shown to be 87.5%. However, in the study done by Abdou et al. maspin expression in BCC was 48%, probably due to the presence of 3 metatypical cases [12]. In their study, 16% and 32% of BCC cases showed weak and moderate expression of maspin, respectively, and no cases had strong maspin expression [12]. We also did not detect strong expression of maspin in any cases, and weak and moderate maspin expressions were found to be present in 34.9% and 39.5% of our cases.

Result of our study was not in favor of any difference between different types of BCC in maspin expression regarding positivity, intensity, and pattern of expression or any association with the age of patient. Also, we did not find any significant correlation between maspin expression and tumor margin involvement.

Maspin is predominantly a soluble cytoplasmic protein and its presence in the nucleus is due to passive diffusion through the nuclear membrane or due to chaperonage to the nucleus [12]. Some studies have revealed that nuclear expression of maspin is associated with better prognosis in various tumors like breast cancer [14], non-small-cell carcinoma of the lung [15], and pancreatic ductal adenocarcinoma [16]. Maspin nuclear expression was also correlated with a lower recurrence rate and a longer disease-free interval after surgery of laryngeal squamous cell carcinoma [17].

We detected nuclear expression of maspin in 59.4% of all cases who expressed this marker and the remaining samples showed only cytoplasmic expression. However, in a study done by Abdou et al. only 33.3% of positive cases had nuclear expression of maspin [12]. The importance of our finding is to be verified since it was previously suggested that nuclear staining of maspin in cutaneous BCC has tumor suppressor role [12]. Abdou et al. detected significant association between nuclear expression of maspin with older age and adenoid variant in cutaneous BCC [12]. However, we did not find any association between nuclear expression of maspin with patient age and tumor margin involvement. We also did not detect any significant difference between adenoid, nodular, and morpheaform subtypes regarding nuclear and cytoplasmic expression of maspin.

Previous studies have shown that expression of ezrin, a member of the ERM (ezrin-radixin-moesin) cytoskeleton-associated protein family, is correlated with poor outcome in many types of human cancers [18]. For example, cytoplasmic expression of ezrin was associated with higher grade, hormonal-receptor negativity, and lymph-node metastases in breast cancer [19]. Ezrin expression was shown to be higher in squamous cell carcinoma compared with less aggressive tumors such as Bowen's disease, actinic keratosis, keratoacanthoma, and seborrheic keratosis [9]. Furthermore, ezrin expression was found to be correlated with metastasis, tumor thickness, progression, and invasion in primary melanomas of the skin [20]. Ezrin overexpression in gastrointestinal stromal tumors was associated with the nongastric location and decreased disease-free survival [10]. Ezrin was expressed in 93% of our cases. Our results are in agreement with previous studies in the fact that intensity of ezrin staining was significantly higher in more aggressive morpheaform BCC compared to adenoid and nodular types. There was no significant association between ezrin expression and patient's age or margin involvement by tumor.

To the best of our knowledge, no relationship between expression of maspin, as a tumor suppressor product, and ezrin, as a marker of tumor progression, has been reported in BCC. No significant association was found between maspin and ezrin expression except in nodular type. There was an inverse correlation between maspin and ezrin expression in this type of periocular tumor.

## 5. Conclusion

We can conclude that maspin is expressed in most cases of periocular BCC regardless of its subtype, while ezrin intensity was higher in morpheaform BCC compared to nodular and adenoid types. There was no association between maspin and ezrin expression except in nodular type, in which inverse correlation exists between maspin and ezrin expression. No correlation was found between maspin and ezrin expression and the age of patient and tumor margin involvement at the time of surgery.

The shortcoming of our study was the limited number of cases. Lack of significant correlation between margin involvement and maspin or ezrin expression had low power to detect clinical significance because of *P* values near 0.05 (*P* = 0.12 and 0.058, resp.). Furthermore, lack of data on clinical outcomes of our patients possibly affects the clinical utility of our results. More cases are needed to highlight the importance of expression of these two tumor markers on diagnosis and outcome of patients with BCC. We also recommend comparison of periocular BCC cases with BCC in other parts of the body regarding maspin and ezrin expression, due to the fact that region of tumor origin may affect its biological and clinical features.

## Figures and Tables

**Table 1:** Clinical and pathological data of different types of periocular BCC.

	Morpheaform BCC (*n* = 10)	Nodular BCC (*n* = 25)	Adenoid BCC (*n* = 8)
Sex (M/F)	5/5	11/14	5/3
Age (mean ± SD)	67.85 ± 19.13	62.5 ± 16.16	57.5 ± 17.57
Age (range)	(26–95)	(31–85)	(26–85)
Involved surgical margin	6 (60%)	10 (40%)	2 (25%)

**Table 2:** Maspin protein expression among different types of periocular BCC.

	Morpheaform BCC (*n* = 10)	Nodular BCC (*n* = 25)	Adenoid BCC (*n* = 8)	Total (*n* = 43)	*P* value
Positivity (%)	8 (80)	17 (68)	7 (87.5)	32 (74.4)	0.63^*^
Intensity					0.82^*^
Negative	2 (20)	8 (32)	1 (12.5)	11 (25.6)	
Weak (%)	5 (50)	8 (32)	2 (25)	15 (34.9)	
Moderate (%)	3 (30)	9 (36)	5 (62.5)	17 (39.5)	
Strong	0	0	0	0	
Pattern					
Cytoplasmic (%)	5 (62.5)	7 (41.2)	1 (12.5)	13 (40.6)	0.25^*^
Cytoplasmic/nucleus (%)	3 (37.5)	10 (58.8)	6 (62.5)	19 (59.4)	0.15^*^

^*^Fisher's exact test.

**Table 3:** Ezrin expression among different types of periocular BCC.

Type	Morpheaform BCC (*n* = 10)	Nodular BCC (*n* = 25)	Adenoid BCC (*n* = 8)	Total (*n* = 43)	*P* value
Positivity (%)	10 (100)	23 (92)	7 (87.5)	40 (93)	0.75^*^
Intensity					<0.001^∗#&^
Weak (%)	0 (0)	15 (60)	3 (37.5)	18 (41.9)	
Moderate (%)	1 (10)	5 (20)	2 (25)	8 (18.6)	
Strong (%)	9 (90)	3 (12)	2 (25)	14 (32.5)	

^*^Fisher's exact test.

^#^Intensity of ezrin expression was significantly higher in morpheaform BCC compared to nodular and adenoid types (*P* < 0.001 and *P* = 0.012, resp.).

^&^No significant difference between nodular and adenoid types (*P* = 0.66).
